# Development of regional pharmacy intravenous admixture services data reporting and analysis platform for enhanced quality control ability

**DOI:** 10.1186/s12913-024-10696-8

**Published:** 2024-02-22

**Authors:** Shaowu Tian, Genyu Xie, Fan Xu, Jun Zhang

**Affiliations:** 1 Graduate School, Kunming Medical University, No. 1168, Chunrong West Road, Yuhua Street, Chenggong District, Kunming, China; 2 Department of Pharmacy, 920 Hospital of Joint Logistics Support Force, No. 212, Daguan Road, Xishan District, Kunming, China; 3 The First Affiliated Hospital of Kunming Medical University, No. 295 Xichang Road, Wuhua District, Kunming, China

**Keywords:** Pharmacy intravenous admixture services, Pharmacy Intravenous Admixture Services Data Reporting and Analysis Platform, PIVAS management

## Abstract

**Background:**

Pharmacy intravenous admixture service (PIVAS) center has emerged as an important department of hospitals as it can improve occupational protection and ensure the safety and effectiveness of intravenous infusions. However, there is little research on the standardized capability and risk evaluation of PIVAS by using modern information technology. In this research, we established Regional Pharmacy Intravenous Admixture Services Data Reporting and Analysis Platform (RPDRAP) to improve quality control ability for PIVAS management. RPDRAP including evaluation matrix for quality control monitoring. The construction of platform is based on guidelines for the Construction and Management of PIVAS and management specifications of PIVAS in China.

**Methods:**

RPDRAP was established in 2018. This platform comprises a data collection system and a data analysis system. The data collection system consists of 67 data items. Data collection relied on online platforms through data acquisition module. The collected data were analyzed using a model with 20 indicators within the data analysis system. Fifteen hospitals, public comprehensive healthcare facilities with more than 500 beds, participated in the platform’s application evaluation.

**Results:**

The study revealed significant differences in PIVAS total score, supervisors, and workload between 2020 and 2022. The platform’s application results demonstrated improvements in personnel management, work efficiency, and infection control within these PIVAS. Although statistical significance was observed in only 8 out of the 25 items, most of the scores showed an increase, with a small portion remaining unchanged and no decline in scores.

**Conclusions:**

This platform can be recommended for PIVAS homogeneous and regional efficient management. The use of this platform not only improves the quality control ability of PIVAS but also enables the management department to quickly grasp the current situation and characteristics of each PIVAS through standardized data collection and analysis.

## Background

Intravenous drug therapy is widely used in clinical treatment in China. The percentage of inpatients receiving intravenous infusion exceeds 80% [1]. However, intravenous administration is considered the most risky method of drug therapy, as it can lead to severe adverse reactions and pose a threat to human life and health. According to the 2021 National Annual Monitoring Report on adverse reactions, 55.3% of adverse reactions are attributed to injection administration, with 90.5% of these adverse reactions specifically related to intravenous administration [2].

Pharmacy intravenous admixture services (PIVAS) is a department within medical institutions that provides specialized technical services for the centralized compounding of intravenous medications for patients. In PIVAS, trained pharmacists offer pharmaceutical services such as intervention and review of intravenous medication prescriptions, compounding and mixing of medications, and participation in the assessment of intravenous infusion usage [3, 4]. Studies have indicated that the implementation of PIVAS can significantly improve the level of rational drug use, enhance infusion safety, and reduce occupational exposure among healthcare workers [5, 6]. Additionally, it has been shown to decrease medication preparation errors and result in cost savings [7]. PIVAS has been widely adopted in developed countries such as the United States, Canada, Australia, New Zealand, and the United Kingdom [8–10]. In China, over 2,000 PIVAS have been implemented. However, it is important to emphasize that PIVAS is a high-risk institution. Inadequate hardware, unreasonable dispensing processes, and chaotic personnel management can lead to large-scale and unforeseen drug accidents. Abdulwahid and Al-Ani. (2020) collected 99 cases from five hospitals. Among these cases, 52 were drug accidents caused by improper use of intravenous injection include drug-drug interaction, drug-disease interaction, and not indicated medication [11]. Curran. (2011) found that infusate contamination can cause infusate-related bloodstream infection and even death [12]. Moyen. et al. (2008) discovered drug errors rank seventh in terms of causes of death [13].

At present, many studies have shown the importance of information management in pharmacy administration. A study by Mazrouei. et al. (2021) showed that standardized data collection is conducive to reduce over-the-counter drug abuse [14]. Meslamani. et al. (2021) showed that pharmacists’ remote intervention through telephone and Internet impact on the medication and clinical outcomes of patients with COVID-19 in rural areas [15]. Abdel-Qader. et al. (2022) showd that online collaborative consultation between pharmacists and doctors significantly reduce people’s resistance to the COVID-19 vaccine [16]. Although various intelligent machines and systems have been implemented in PIVAS, including labeling systems, infusion sequence annotation systems, review prescription databases, and so on [17–19], the utilization of this equipment only addresses workload challenges and helps mitigate human errors in specific aspects of the workflow. At present, there is little research on the standardized capability and risk evaluation of PIVAS by using modern information technology. In this research, we established the Regional Pharmacy Intravenous Admixture Services Data Reporting and Analysis Platform (RPDRAP), based on guidelines for the Construction and Management of PIVAS and management specifications of PIVAS in China, to improve quality control ability for PIVAS management.

## Methods

### Platform structure

RPDRAP consists of two systems: the data collection system and the data analysis system. The data collection system comprises a data acquisition module and a data audit module. The data collection module is displayed in the form of an online questionnaire for users to fill in the relevant information including basic information, hardware management, personnel management, work efficiency, information management, and infection control management aspects of 67 data collection items of PIVAS (Table 1). When the data reporter of PIVAS submits the form online every quarter, the system will verify the integrity and validity of the information. Once approved by the experts using the data audit module, the data will be stored in the SQL server database.

**Table 1:** Data collection items of data collection system

Aspect	Item	Unit	Data type	Data item number	Item	Unit	Data type	Data item number
**Basic Information**	Name of medical institution		String	A01	Position		String	A07
	Address		Varchar	A02	Hospital grade		[character]	A08
	Legal representative		String	A03	Postal code		Integer	A09
	Director of Pharmacy Department		String	A04	Director of medical institution		String	A10
	Fix telephone number		Integer	A05	Phone number		Integer	A11
	Reporter		string	A06	Start operating time		Time	A12
**Hardware management**	working area	m²	Float	B01	Number of fans	Unit	Integer	B07
	Total number of beds	Bed	Integer	B02	Number of horizontal laminar flow console	Unit	Integer	B08
	Number of hospital wards	Unit	Integer	B03	Rest area	m²	Float	B09
	The number of beds provided (temporary prescription)	Unit	Integer	B04	The number of beds provided by PIVAS	Bed	Integer	B10
	Number of clean bench	Unit	Integer	B05	The number of beds provided (long-term prescription)	Bed	Integer	B11
	Number of air conditioners	Unit	Integer	B06	Number of biosafety cabinets	Unit	Integer	B12
**Personnel management**	Number of assistant staff	People	Integer	C01	Number of nurses	People	Integer	C06
	Number of training at above the municipal level	Times	Integer	C02	Number of training in hospital	Times	Integer	C07
	Number of people trained at or above the municipal level	People	Integer	C03	Number of people trained in hospital	People	Integer	C08
	Number of beds provided by PIVAS	Bed	Integer	C04	Title of director		String	C09
	Degree of director		String	C05	Number of pharmacists	People	Integer	C10
**Work efficiency management**	Total amount of infusion prepared	Bag	Integer	D01	General drug dispensing amount	Bag	Integer	D11
	Quantity of antibiotics dispensed	Bag	Integer	D02	Parenteral nutrition dispensing amount	Bag	Integer	D12
	Quantity of anticancer drug dispensed	Bag	Integer	D03	Average number of infusion preparations per person	Bag	Integer	D13
	Daily number of prescriptions reviewed per person	Piece	Integer	D04	Total amount of prescription	Piece	Integer	D14
	Daily average dispensing quantity for long-term prescription	Bag	Integer	D05	Daily average dispensing quantity for temporary prescription	Bag	Integer	D15
	Number of unreasonable prescription	Piece	Integer	D06	Proportion of unreasonable prescription	%	Float	D16
	Disposal of unreasonable prescription (packing or returning)		[Character]	D07	Number of unreasonable prescription that physician agree to modify	Piece	Integer	D17
	Number of errors in displaying	Bag	Integer	D08	Number of prescription review errors	Piece	Integer	D18
	Number of disposable syringes used	Unit	Integer	D09	Number of wrong dispensing	Bag	Integer	D19
	Total number of infusion distribution	Bag	Integer	D10				
**Infection control management**	Comprehensive cleaning frequency	Times	Integer	E01	Temperature qualification rate	%	Float	E07
	Humidity qualification rate	%	Float	E02	Rate of qualified pressure	%	Float	E08
	Microorganisms qualification rate	%	Float	E03	Number of cleanliness detection	Times	Integer	E09
	Times of primary air filter maintenance	Times	Integer	E04	Frequency of replacement of disinfectant		Integer	E10
	Times of medium efficiency air filter maintenance	Times	Integer	E05	Disinfection method (ethanol, ultraviolet or other)		String	E11
	Times of high efficiency air filter maintenance	Times	Integer	E06				
**Information management**	Automatic equipment		[Character]	F01	Configuration of clinical records management information system		Boolean	F03
	Configuration of information systems		Boolean	F02				

**Fig. 1:**
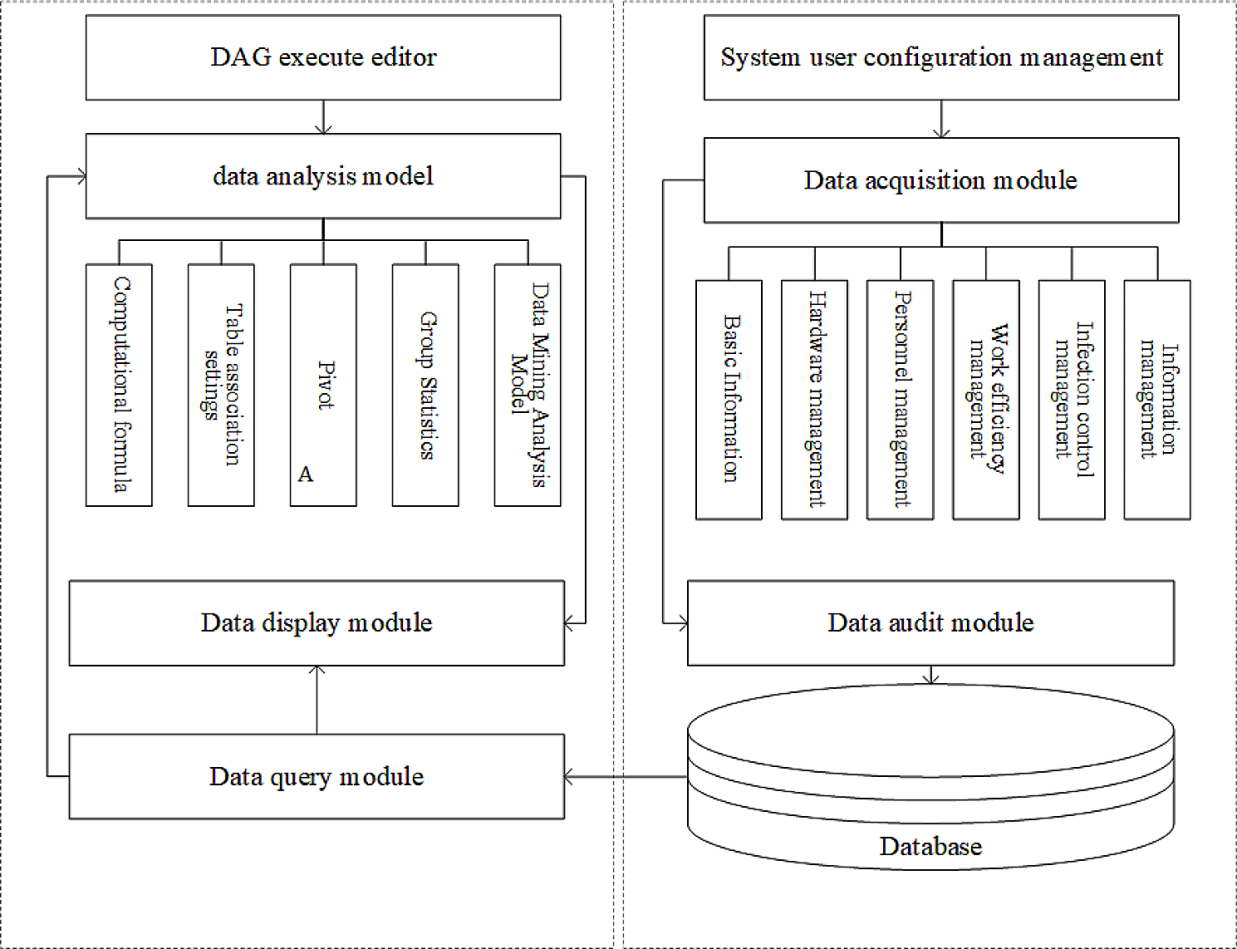
Framework of RPDRAP

The data analysis system comprises a directed acyclic graph(DAG)execute editor, data query model, data analysis module, and data display module. DAG execute editor is used to implement system operations through visualization. The data analysis module consists of functions of computational formula, Table association settings, pivot, group statistics, and data mining analysis method. We had created an evaluation matrix which is composed of 20 scoring indicators and developed by an expert group from the Yunnan Pharmaceutical Association and the Yunnan Pharmaceutical Administration Quality Control Center are shown in Table 2. The evaluation results of each PIVAS are obtained through this model and presented in the form of radar charts and score Tables through data display module. The framework of RPDRAP is shown in Fig. 1.

**Table 2:** evaluation matrix

Indicators	Scoring requirements	Score calculation method	Data source
***Hardware management***		***11***	
Rationality of working area	Average daily deployment is less than 1000 bags, the working area shall not be less than 300 m^2^. The Average is 1001 ~ 2000 bags, working area should be 300 ~ 500m^2^. The number of dispensing infusions per day is 2001 ~ 3000 bags, working area should be 500 ~ 650m^2^. The Average is more than 3000 bags, the working area will be increased by 50m^2^ for every 500 bags added.	Score: 5 points. Scoring criteria: when meeting scoring requirements, 5 points will be given.	B01, D05 + D15 (D05 + D15: average daily deployment)
Auxiliary functional area	PIVAS has a Secondary drug warehouse, material storage area, drug outsourcing area, transfer box/transfer vehicle storage area, and meeting and teaching lounge.	Score: 1 point. Scoring criteria: when meeting the scoring requirements, 1 point will be given.	B09
Rationality of workbench quantity for service bed	One double operator console should be equipped for every 100 beds at least.	Score: 5 points. Scoring criteria: when meeting the scoring requirements, 5 points will be given.	B05 + B08 + B12 > B02 > B10, (B05 + B08 + B12: double operator console)
***Personnel management***		***14***	
Staff training	Personnel of PIVAS is relatively fixed, pass training and examination of professional knowledge and technical operation specifications, and receive regular continuing medical education.	Score: 5 points. Scoring criteria: when number of training sessions per person is greater than 1, 5 points will be given. When the number is between 1 and 0.2, 2 points will be given. When the number is less than 0.2, 0 points will be given.	(C02 + C07)/(C03 + C08) (C02 + C07: training times, C03 + C08: number of trainers)
Personnel types	Pharmacists.	Score: 5 points. Scoring criteria: when all staff are pharmacists, 5 points will be given. When proportion of pharmacists is between 80% and 50%, 4 points will be given. When proportion of nurser is more than 50%, 2 points will be given. All staff are nurses, 0 score will be given.	C06, C10
Director	The person in charge of PIVAS shall be qualified for professional and technical posts above the intermediate level.	Score: 4 points. Scoring criteria: when title and degree of director are above of supervising pharmacist and bachelor’s degree, 4 points will be given. When title and educational level are supervising pharmacist and bachelor’s degree below, 2 points will be given.	C09, C05
***Work efficiency management***		***49***	
Type of infusion	PIVAS should provide intravenous drug dispensing services, including parenteral nutrition, anticancer drugs, antibiotics drugs, and general drugs	Score: 10 points. Scoring criteria: when 4 types of drugs are dispensed, 4 points will be given. When parenteral nutrition or anticancer drugs are dispensed, 3 points will be given. When antibiotics drugs or general drugs are dispensed, 2 points will be given.	D02, D03, D11, D12
Average daily deployment	According to an average number of prepared infusions daily of PIVAS in Yunnan Province in the past few years.	Score: 5 points. Scoring criteria: when average daily deployment more than the average, 5 points will be get. Average daily deployment is between two-thirds and one of average,4 points will be given. Average daily deployment is between two-thirds and one-thirds of average,3 points will be given. Average daily deployment is less than one-thirds of average, 2 points will be given.	D05 + D15 (D05 + D15: average daily deployment)
Daily bed infusion quantity	Based on the average number of daily infusions per sickbed of PIVAS in Yunnan Province in the past few years.	Score: 3 points. Scoring criteria: daily bed infusion quantity is less than average,3 points will be given. The quantity is more than average, 1 point will be given.	D10/B10 (D10/B10: daily bed infusion quantity)
Usage of syringe	According to the average number of dispensing infusion groups for each syringe of PIVAS in Yunnan Province in the past few years.	Score: 5 points. Scoring criteria: the average number of dispensing infusion groups for each syringe is less than average,5 points will be given. The average number is more than average, 4 points will be given.	D01/D09 (D01/D09: the average number of dispensing infusion groups for each syringe)
Daily infusion allocation quantity per person	80–100 bags.	Score: 5 points. Scoring criteria: daily infusion allocation quantity per person is less than 100,5 points will be given.	D13
Temporary order deployment rate	The number of temporary medical orders allocated is proportional to the PIVAS service capacity.	5 points. Scoring criteria: temporary order deployment rate is higher than 5%, 5 points will be given. The rate is between 5% and 3%, 3 points will be given. The rate is lower than 3%, no point.	D15/D05 (D15/D05: temporary order deployment rate)
Daily number of prescriptions reviewed per person	To ensure the quality and efficiency of the audition, reviewed prescriptions per person per day are less than 500 medical orders in PIVAS.	Score: 6 points. Scoring criteria: daily number of prescriptions reviewed per person is less than 500, 6 points will be given. The number is between 800 and 500, 3 points will be given. The number is greater than 800,0 points will be given.	D04
Unreasonable prescription and disposition	Based on the average rate of unreasonable prescription and disposition of PIVAS in Yunnan Province in the past few years.	Score: 5 points. Scoring criteria: the rate of unreasonable prescription is lower than the average and disposition rate is higher than the average, 5 points will be given. The rate and disposition rate are more than the average, 3 points will be given. The rate is lower than the average and disposition rate is higher than average, 1 point will be given.	D17/D06,D16 (D17/D06: disposition rate of unreasonable prescription)
Error control rate	The error rate of dispensed infusion of PIVAS in Yunnan Province in the past few years.	Score: 5 points. Scoring criteria: error control rate is less than the average, 5 points will be given. Error control rate exceeds 30% of the average, 2 points will be given.	D08 + D18 + D19/D01 (D08 + D18 + D19:error qualities of infusion preparation)
***Infection control management***		***13***	
Comprehensive cleaning frequency	We shall clean and disinfect the workbench and floor at least every day. Fully clean PIVAS at least once a week.	Score: 5 points. Scoring criteria: comprehensive cleaning frequency is at least once a week, 5 points will be given. The frequency is once between a week and two weeks, 3 points will be given. The frequency is once a month, 1 point will be given.	E01
Air filter cleaning and maintenance	Clean and maintain air filters at least annually.	Score: 5 points. Scoring criteria: completing cleaning and maintenance of all air filters within one year, 5 points will be given. Completing the cleaning and maintenance of one to two types of air filters within one year, 3 points will be given. Completing the cleaning and maintenance of no air filters within one year,0 points will be given.	E04, E05, E06
Disinfection method	At least two disinfectants are selected for disinfection in PIVAS.	Score: 3 points. Scoring criteria: two or more disinfection methods are used in PIVAS, 3 points will be given. Only one disinfection method is used in PIVAS,0 points will be given.	E11
***Information management***		***13***	
Degree of informatics	The more intelligent systems and instruments, the higher the degree of informatization.	Score: 8 points. Scoring criteria: when PIVAS has configuration of information systems, 3 points will be given. When PIVAS has clinical records management information system, 2 points will be given. PIVAS has two types of automatic equipment, 2 points will be given.	F01, F02, F03
Tracing control	Determine based on whether PIVAS has corresponding intelligent instruments, such as drug dispensing scanner, vertical flow clean bench, etc.	Score: 5 points. Scoring criteria: tracing control is higher 2, 5 points will be given. Tracing control is between 1 and 2, 3 points will be given. Tracing control is between 1 and 0.5, 2 points will be given. Tracing control is between 0.5 and 0.1, 1 point will be given.	F02/B05 + B08 + B12 (B05 + B08 + B12:double operator console)
***Total scores***		***100***	

Note: The data source is from Table 1, and only data item number is referenced in this Table

The evaluation matrix was verified by an expert group from the Yunnan Pharmaceutical Association and the Yunnan Pharmaceutical Administration Quality Control Center.

### Platform application

We had carried out the research and application of this platform in Yunnan Province, China. Since its launch in 2020, an increasing number of PIVAS had participated in the platform’s application. In 2020, there were 28 participating PIVAS, followed by 55 in 2021 and 71 in 2022.

Through this platform, PIVAS regularly reports information related to its operations. Additionally, PIVAS could easily access their own PIVAS scores for the 20 indicators, as well as the average, maximum, and minimum scores of PIVAS in the region.

### Study subjects

To evaluate whether the application of the platform promoted the standardized construction and development of PIVAS, the following inclusion criteria for study subjects were formulated: (1) The subjects under investigation were second-level and above public hospitals (hospitals of county-level and above) in Yunnan Province. (2) The medical institutions in Yunnan Province had implemented PIVAS and had reported data in RPDRAP. (3) Complete data for the years 2020, 2021, and 2022 were submitted in RPDRAP. Fifteen PIVAS met the inclusion criteria, and their three-year operational data were selected as the research objects.

### Statistical analyses

Due to the non**-**normal distribution of the data tested by the normality checking tested Shapiro-Wilk test, we analyzed the data of 20 indicators in five aspects of 15 PIVAS in the past three years with the generalized estimation equation and pairwise comparison. SPSS statistical software (SPSS 24) was used to analyze the data.

## Result

**Fig. 2:**
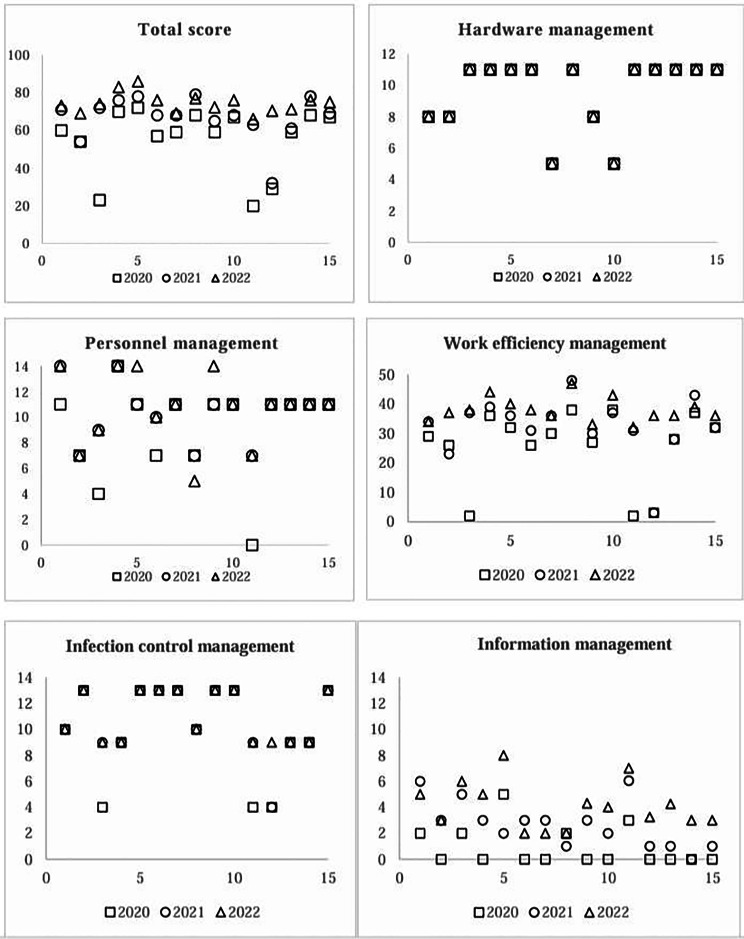
PIVAS total score of 15 hospital scatter diagram

The analysis results of 20 indicators across five aspects over three years for the 15 PIVAS are presented in Tables 3 and 4. The scores for the total and each aspect of the 15 PIVAS are displayed in Fig. 2. Over three years, significant improvements were observed in 8 indicators of five aspects (see Table 4).

**Table 3:** Mean and standard deviation of 20 indicators of 15 PIVAS in three years

Indicator	M + SD(2020)	M + SD(2021)	M + SD(2022)
***Hardware management***	9.60 ± 2.22	9.60 ± 2.22	9.60 ± 2.22
Rationality of working area	4.00 ± 1.46	4.00 ± 1.46	4.00 ± 1.46
Auxiliary functional area	1.00 ± 0.00	1.00 ± 0.00	1.00 ± 0.00
Rationality of workbench quantity for service bed	4.60 ± 1.06	4.60 ± 1.06	4.60 ± 1.03
***Personnel management***	***9.20 ± 3.57***	***10.40 ± 2.16***	***10.67 ± 2.77***
Director	2.13 ± 2.07	2.13 ± 2.07	2.13 ± 2.07
Personnel types	2.80 ± 2.01	3.27 ± 1.75	3.60 ± 1.55
Staff training	2.13 ± 2.00	3.53 ± 1.96	4.33 ± 1.76
***Work efficiency management***	***25.73 ± 12.79***	***32.53 ± 10.18***	***37.93 ± 4.15***
Type of infusion	7.20 ± 3.95	8.33 ± 2.72	9.20 ± 1.73
Average daily deployment	3.87 ± 1.30	3.87 ± 1.30	3.87 ± 1.30
Daily bed infusion quantity	2.33 ± 1.23	2.47 ± 0.83	2.60 ± 0.63
Usage of syringe	0.93 ± 1.94	1.20 ± 2.08	2.87 ± 2.13
Daily infusion allocation quantity per person	1.80 ± 1.21	3.07 ± 1.71	3.80 ± 1.52
Temporary order deployment rate	1.67 ± 2.44	1.67 ± 2.44	1.67 ± 2.44
Daily number of prescriptions reviewed per person	3.40 ± 2.50	4.60 ± 2.23	5.80 ± 0.77
Unreasonable medical order and disposition	2.07 ± 2.49	3.67 ± 2.89	4.13 ± 1.64
Error control rate	2.47 ± 2.20	3.67 ± 1.99	4.00 ± 1.73
***Infection control management***	***10.00 ± 3.53***	***10.67 ± 2.64***	***11.00 ± 1.96***
Comprehensive cleaning frequency	4.00 ± 2.07	4.67 ± 1.29	5.00 ± 0.00
Air filter cleaning and maintenance	3.40 ± 2.03	3.40 ± 2.03	3.40 ± 2.03
Disinfection method	2.60 ± 1.06	2.60 ± 1.06	2.60 ± 1.06
***Information management***	***0.93 ± 1.53***	***3.60 ± 1.50***	***5.05 ± 1.51***
Degree of informatics	0.00 ± 0.00	2.67 ± 0.82	4.12 ± 1.06
Tracing control	0.93 ± 1.53	0.93 ± 1.53	0.93 ± 1.53
***Total scores***	***55.47 ± 17.19***	***66.8 ± 11.62***	***74.25 ± 5.32***

Note: “M” and “SD” respectively represents mean and standard deviation

**Table 4:** Pairwise comparisons analysis of 20 indicators of 15 PIVAS in three years

Indicator		Standard Error	*P*
***Hardware management***	***20vs21***	***0.00***	***1.00***
	***20vs22***	***0.00***	***1.00***
	***21vs22***	***0.00***	***1.00***
Rationality of working area	20vs21	0.00	1.00
	20vs22	0.00	1.00
	21vs22	0.00	1.00
Auxiliary functional area	20vs21	0.00	1.00
	20vs22	0.00	1.00
	21vs22	0.00	1.00
Rationality of workbench quantity for service bed	20vs21	0.00	1.00
	20vs22	0.00	1.00
	21vs22	0.00	1.00
***Personnel management***	***20vs21***	***0.56***	0.03 *
	***20vs22***	***0.60***	0.01*
	***21vs22***	***0.31***	***0.38***
Director	20vs21	0.00	1.00
	20vs22	0.00	1.00
	21vs22	0.00	1.00
Personnel types	20vs21	0.33	0.16
	20vs22	0.33	0.16
	21vs22	0.00	1.00
Staff training	20vs21	0.45	0.08
	20vs22	0.58	< 0.001**
	21vs22	0.53	0.008**
***Work efficiency management***	***20vs21***	***2.13***	0.01*
	***20vs22***	***2.49***	< 0.001**
	***21vs22***	***2.17***	0.01*
Type of infusion	20vs21	0.66	0.19
	20vs22	0.93	0.03*
	21vs22	0.76	0.14
Average daily deployment	20vs21	0.00	1.00
	20vs22	0.00	1.00
	21vs22	0.00	1.00
Daily bed infusion quantity	20vs21	0.26	0.46
	20vs22	0.32	0.22
	21vs22	0.21	0.35
Usage of syringe	20vs21	0.00	1.00
	20vs22	0.56	< 0.001**
	21vs22	0.56	< 0.001**
Daily infusion allocation quantity per person	20vs21	0.47	0.03*
	20vs22	0.46	< 0.001*
	21vs22	0.48	0.10
Temporary order deployment rate	20vs21	0.00	1.00
	20vs22	0.00	1.00
	21vs22	0.00	1.00
Daily number of prescriptions reviewed per person	20vs21	0.68	0.08
	20vs22	0.60	< 0.001*
	21vs22	0.46	0.03*
Unreasonable prescriptions and disposition	20vs21	0.54	0.02*
	20vs22	0.56	< 0.001*
	21vs22	0.49	0.35
Error control rate	20vs21	0.36	0.01*
	20vs22	0.58	0.01*
	21vs22	0.52	0.20
***Infection control management***	***20vs21***	***0.44***	***0.13***
	***20vs22***	***0.52***	***0.05***
	***21vs22***	***0.32***	***0.30***
Comprehensive cleaning frequency	20vs21	0.44	0.13
	20vs22	0.52	0.05
	21vs22	0.32	0.30
Air filter cleaning and maintenance	20vs21	0.00	1.00
	20vs22	0.00	1.00
	21vs22	0.00	1.00
Disinfection method	20vs21	0.00	1.00
	20vs22	0.00	1.00
	21vs22	0.00	1.00
***Information management***	***20vs21***	***0.26***	< 0.001*
	***20vs22***	***0.35***	< 0.001*
	***21vs22***	***0.22***	< 0.001*
Degree of informatics	20vs21	0.20	< 0.001**
	20vs22	0.33	< 0.001**
	21vs22	0.22	< 0.001**
Tracing control	20vs21	0.00	1.00
	20vs22	0.00	1.00
	21vs22	0.00	1.00
Total scores	20vs21	3.11	< 0.001**
	20vs22	3.40	< 0.001**
	21vs22	2.39	< 0.001**

Note: “* ”means *P* < 0.05; “* * ”means P < 0.01

The indicators of “type of infusion”, “the number of comprehensive cleaning times”, and “the number of prescriptions per person per day” improved significantly, and the final score was close to the highest score.

The indicators of “usage of syringe (P < 0.001)”, “daily infusion allocation quantity per person (P < 0.05)”, “unreasonable medical order and disposition (P < 0.001)”, “work efficiency management (P < 0.01)” and “information management (P < 0.01)” were significantly improved, but further improvements are needed to meet the standard requirements.

The indicators of “staff training (P < 0.01)”, “daily number of prescriptions reviewed per person (P < 0.05)”, “error control rate (P = 0.01)”, “degree of informatics (P < 0.001)” and “unreasonable medical order disposition (P < 0.05)” were significantly improved, and have been more than 80% of the total score.

Although the scores of “air filtration”, “daily bed infusion quantity”, “average daily deployment”, “personnel management”, and “infection control management” showed an upward trend but there was no significant difference in scores.

The indicators of “rating of work area”, “average daily deployment”, “all air filter cleaning and maintenance” and “disinfection method” haven’t changed for three years and the average score rate exceeded 60% of standard requirements.

The indicators of “director”, “temporary order deployment rate”, and “tracking control” have no change and the average score rate was less than 60% of standard requirements.

## Discussion

At present, quality control has attracted much attention by PIVAS managers. There have been many studies on this research area, but majority of studies more focused on improvement of quality control using a particular innovative technology or equipment. Deng. et al. (2022) explored the development of automatic auxiliary dispensing equipment in PIVAS to improve the work efficiency, and reduce the drug risk caused by dispensing errors [20]. Gao. et al. (2020) found that implementation of lean had positive results, which improved the efficiency of the operation, reduced the work start time and the amount of staff, and improved clinical satisfaction [21]. Yang. et al. (2023) investigated the emotional disorders including depression and anxiety among staff of PIVAS [3]. The result show depression and anxiety are common among PIVAS leaders and staff working in hospitals in China. Hospitals should implement measures to improve the mental health of PIVAS leaders and staff. Chen. et al. (2021) analyzed the current situation of personnel training and scientific research regarding PIVAS [22]. The findings indicated that the training content for PIVAS personnel in China was relatively comprehensive, but the areas of management tools, career development, and scientific research training were comparatively deficient, resulting in very low scientific research output. However little research focused on how to evaluate the capability of quality control of existing PIVAS and research in this area is considered to be of great importance for the management of PIVAS.

In this research, we established RPDRAP. Through the platform application, PIVAS managers can not only better understand the requirements of each indicator, but also obtain the highest, lowest, average, and own scores of each indicator within the region. In this way, PIVAS managers can more accurately manage and control the problems that exist in PIVAS. The effect of system application was shown in the results. Due to the high cost of hardware management including the work area, biosafety cabinets, and air conditioning equipment, it is difficult and time-consuming to update. This result can be interpreted as the score for hardware management of the 15 PIVAS has remained unchanged over the past three years. Once the hardware management of PIVAS are completed, there is no margin for changes. PIVAS included in the statistical analysis was the first batch to use the platform, the hardware construction standards were not uniform at that time. In terms of the hardware score of PIVAS added to the platform application in the later stage, it has made significant progress.

Furthermore, over the past three years, the item scores for 5 indicators such as directorship, temporary order deployment quantity, and so on have consistently remained relatively low. The average score of director was 2.13 points, only achieving 53% of the score for this item. County-level hospitals generally have lower scores and remaining unchange in this item. This indicates that the professional and technical level of PIVAS professionals at the county level needs to be improved, and this improvement will take a long time. The average score of temporary order deployment quantity was 1.67 points, only achieving 33.4% of the score for this item. The temporary order deployment quantity requires more professional technical personnel for PIVAS, which is also the reason for the limited improvement and remaining unchanged. The average score of usage of syringe was 2.87 points, only achieving 57.4% of the score for this item. The reuse of syringes in the formulation of similar drugs can save costs.

## Conclusion

The safety of intravenous infusion is very important to the treatment outcome, and the safety of PIVAS depends on many aspects. Such as clean workbench, high-quality disinfection equipment, and high-quality staff. RPDRAP is the first PIVAS unified data management platform in China. The use of this platform enables different PIVAS in the region to compare their management measures with each other, which not only enhances the quality control ability of PIVAS but also enables the management department to understand the current situation and characteristics of each PIVAS timely and comprehensively through standardized data, which provides strong evidence for evaluating the service capability of PIVAS.

## Strengths and limitations

RPDRAP is the first PIVAS unified data management platform in China. Using this platform PIVAS can systematically and quickly find its own shortcomings by comparing the management and capacity building of PIVAS in the region.

Now the platform is only used within Yunnan Province. We hope it can be promoted and applied to a wider range of regions. Furthermore, the evaluation matrix of the analysis system should be further expanded and improved based on the progress of research on quality control.

## Data Availability

The datasets used and analyzed during the current study are available from the corresponding author upon reasonable request.
